# Major evolutionary transitions and innovations: the tympanic middle ear

**DOI:** 10.1098/rstb.2015.0483

**Published:** 2017-02-05

**Authors:** Abigail S. Tucker

**Affiliations:** Department of Craniofacial Development and Stem Cell Biology, King's College London, Floor 27 Guy's Hospital, London Bridge, London SE1 9RT, UK

**Keywords:** middle ear, mammal evolution, tympanic membrane, bulla, jaw joint

## Abstract

One of the most amazing transitions and innovations during the evolution of mammals was the formation of a novel jaw joint and the incorporation of the original jaw joint into the middle ear to create the unique mammalian three bone/ossicle ear. In this review, we look at the key steps that led to this change and other unusual features of the middle ear and how developmental biology has been providing an understanding of the mechanisms involved. This starts with an overview of the tympanic (air-filled) middle ear, and how the ear drum (tympanic membrane) and the cavity itself form during development in amniotes. This is followed by an investigation of how the ear is connected to the pharynx and the relationship of the ear to the bony bulla in which it sits. Finally, the novel mammalian jaw joint and versatile dentary bone will be discussed with respect to evolution of the mammalian middle ear.

This article is part of the themed issue ‘Evo-devo in the genomics era, and the origins of morphological diversity’.

## 1. Multiple origins of the tympanic ear

**Figure 1.**
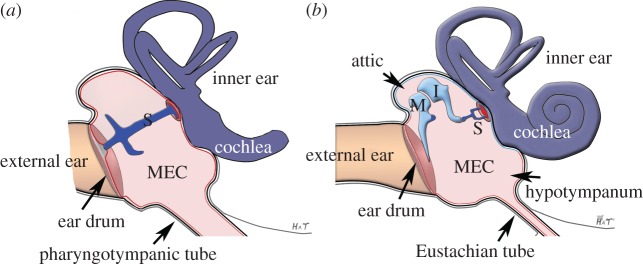
Anatomy of the middle ear. (*a*) Schematic of a sauropsid (bird, lizard) middle ear with a single ossicle spanning the middle ear cavity. (*b*) Schematic of a mammalian middle ear with three ossicles in a chain within the cavity. Origin of ossicles: Light blue denotes first arch neural crest derived tissue. Dark blue denotes second arch neural crest derived tissue. Red denotes mesoderm-derived tissue (stapes footplate). S, stapes; M, malleus; I, incus; MEC, middle ear cavity.

In modern amniotes (birds, reptiles and mammals), the middle ear comprises an air-filled space known as a tympanic ear. Sound travels across this air-filled space via bony ossicles. Formation of a tympanic ear was a key innovation to solve the problems of a mismatch in impedance between air and tissue encountered with the transition from water to land. The tympanic middle ear enables detection of sound pressure by transforming sound energy in air to fluid motion in the inner ear. A tympanic ear is also present in most anurans (frogs and toads), although the Gymnophiona (such as the legless caecilians) and Caudata (salamanders) do not have tympanic ears. Some earless anurans are also found, where the body walls, mouth and lungs have been shown to serve as a route of sound transfer to the inner ear [1,2]. In reptiles and birds (the sauropsids), the middle ear space houses a single suspended ossicle, known as the stapes in reptiles and the columella in birds (figure 1*a*). Neural crest grafting experiments in the chick have shown that the columella is derived from the second pharyngeal arch, with the exception of its base, which sits in the otic capsule and is derived, like most of the capsule, from mesoderm (see red base shown in figure 1*a*) [3,4]. Ablation of the developing otic capsule leads to a defect in the formation of the base of the columella in chick, turtle and urodeles, suggesting that the columella has a similar dual origin in reptiles and amphibians [5–8]. This single ossicle bridges the gap between the tympanic membrane (ear drum) and the inner ear. By contrast, mammals have a chain of three ossicles, the malleus, incus and stapes (figure 1*b*). The stapes is homologous to the stapes/columella in reptiles and birds and, in keeping with this homology, genetic labelling experiments in the mouse have shown that it is largely derived from the second pharyngeal arch, except for the part that sits in the oval window of the otic capsule which is again mesoderm derived (see red base of footplate shown in figure 1*b*) [9]. The malleus and incus, in contrast, are largely first arch derived [10,11]. Figure 1 highlights the origin of the ossicles in sauropsids and mammals.

The arrangement of reptile, bird and mammalian ears, all with ossicles suspended within an air-filled space was originally interpreted as the tympanic ear being ancestral, with stem amniotes predicted to have a very similar arrangement to that observed in modern reptiles. This led to papers reflecting on how a functional tympanic reptilian middle ear could have been transformed into the mammalian middle ear [12,13]. It is now widely accepted that the stem amniote did not have a tympanic middle ear and therefore tympanic middle ears evolved independently across the different amniote groups during the Early Triassic [14,15]. This relatively recent interpretation comes from the fact that in fossils of early stem amniotes a robust large stapes, derived from the hyomandibular, is observed with no evidence for a role in airborne sound. Instead the stapes appears to have played a role in stabilizing the skull during biting. As skulls evolved, new connections developed, freeing the stapes from this structural role and leading to the linking of the stapes to the inner ear for a role in hearing. In all amniotes, except the mammals, a single ossicle tympanic ear evolved, while in mammals a three ossicle ear formed instead, without the need for a transition from a single ossicle tympanic ear to a three ossicle ear. It has been proposed that the three ossicle middle ear may also have evolved more than once, including independently in the lineages leading to the monotremes (egg-laying mammals) and therian mammals (placentals and marsupials) [16]. This would mean that the middle ears of mammals such as the platypus would not be homologous to the ears of a mouse, or ourselves. Whether this is the case, however, has been debated and more data are probably needed to clarify the situation (discussed in [17]). The three ossicle middle ear is thought to be better adapted for the transmission of high frequencies, allowing the ultrasonic hearing typical of many mammals.

## 2. Independent origin of the tympanic membrane (ear drum) and external ear canal

Although an independent origin of the tympanic middle ear has only recently been agreed it was originally proposed by Gaupp [18] based on comparative embryology. Gaupp suggested that an independent origin was the only way of explaining differences in the course of the chorda tympani (a branch of the facial nerve that runs through the middle ear), and the position of the tympanum (the air-filled cavity) with respect to Meckel's cartilage. This view was refuted by Goodrich [19] but was supported by later comparative analysis [20]. An independent evolution of the tympanic ear and therefore the tympanic membrane (ear drum) has been recently supported by developmental biology. If modern mammals, reptiles and birds evolved from a common ancestor with a tympanic ear it would be assumed that the external ear canal and tympanic membrane would form in the same way in the different groups. In fact, the external ear canal forms at different positions within the head in birds (chick) and mammals (mouse), with the tympanic membrane being supported by the quadrate (an upper jaw element) in birds and reptiles while being supported by the tympanic ring/ectotympanic (a lower jaw element) in mammals [18]. To explain the different positions of the tympanic membrane in mammals and sauropsids, Westoll [13] proposed that a ventral diverticulum of the middle ear cavity (the recess mandibularis) grew ventrally to form a mammal-specific tympanic membrane. The mammalian membrane was therefore assumed to form from a dorsal part, corresponding to the reptile membrane, and a ventral part that was a novel structure unique to mammals [13]. In reptiles, the tympanic ring which supports the ear drum is homologous to the angular, while the associated gonial is homologous to the prearticular. During development, this homology is reflected in the way these bones form (membranous ossification), their relative position with respect to the other skeletal elements of the jaw/ear, the relative timing of their development and conserved gene expression [21]. In reptiles and birds, the proximally positioned angular and prearticular are associated with the support of the lower jaw, while in mammals these bones have shifted into the middle ear. This change of function from jaw bone to ear membrane support was triggered by the role of lower jaw support in mammals being taken over by a single bone, the dentary.

The external ear canal in the chick forms above the forming jaw joint, as indicated by expression of *Bapx1*; however, in the mouse the ear canal forms below this expression domain [22]. Different positions of the ear canal in mammals and sauropsids have previously been suggested based on morphology [23]. This difference in position was recently highlighted using genetic manipulation of mouse and chick embryos. *Endothelin* signalling has been shown to be important for patterning of the lower jaw. When *endothelin* signalling is disrupted, *Dlx5/6* expression is inhibited, lower jaw identity is lost and the tissue develops as a mirror image upper jaw [24]. The external ear and tympanic membrane are also lost, as would be expected given that these structures are associated with the lower jaw. A similar loss of lower jaw structures and mirror image duplication of upper jaw elements is observed when endothelin signalling is inhibited in the chick; however, strikingly in this case the ear canal is not lost but is expanded, with the formation of a duplicated tympanic membrane associated with a duplicated columella [22]. In addition, it is very clear from an analysis of the development of the external ear canal that the process of forming a canal is very different in the chick and mouse. In the chick, the canal is formed by an invagination of the surface epithelium [22], whereas in the mouse and humans, the canal is thought to form by elongation of a solid plug of cells (the auditory meatal plug) which later cavitates to generate the canal [25,26]. These differences may relate to the different position of the tympanic membrane, which is much more deeply positioned in mammals. Given the different mode of development of the external ear canal in chicks and mice, and the differences in skeletal support of the tympanic membrane, it appears apparent that these structures are not homologous.

The external ear canal is classically thought to develop at the cleft between the first and second pharyngeal arch [27]. The cleft was thought to invaginate in and meet the first arch pouch invaginating on the inside of the head, with the tympanic membrane forming in between these two structures. The tympanic membrane is therefore derived classically from three germ layers, ectoderm on the outside facing the external ear canal, endoderm on the inside facing the middle ear cavity, with a thin layer of neural crest derived mesenchyme sandwiched in between. In fact, it has recently been shown that the ear canal in mice does not develop as an extension of the first pharyngeal cleft but forms more rostrally, solely within the first pharyngeal arch [28]. The canal therefore forms within a *Hoxa2*-negative mesenchyme. In keeping with this, duplication of first arch structures in the second arch, as observed in the *Hoxa2* knockout mouse, includes duplication of the external ear canal [29]. It would therefore be interesting to assess whether the non-mammalian external ear canal also forms in a region distinct from the first pharyngeal cleft.

## 3. Making an air-filled space

**Figure 2.**
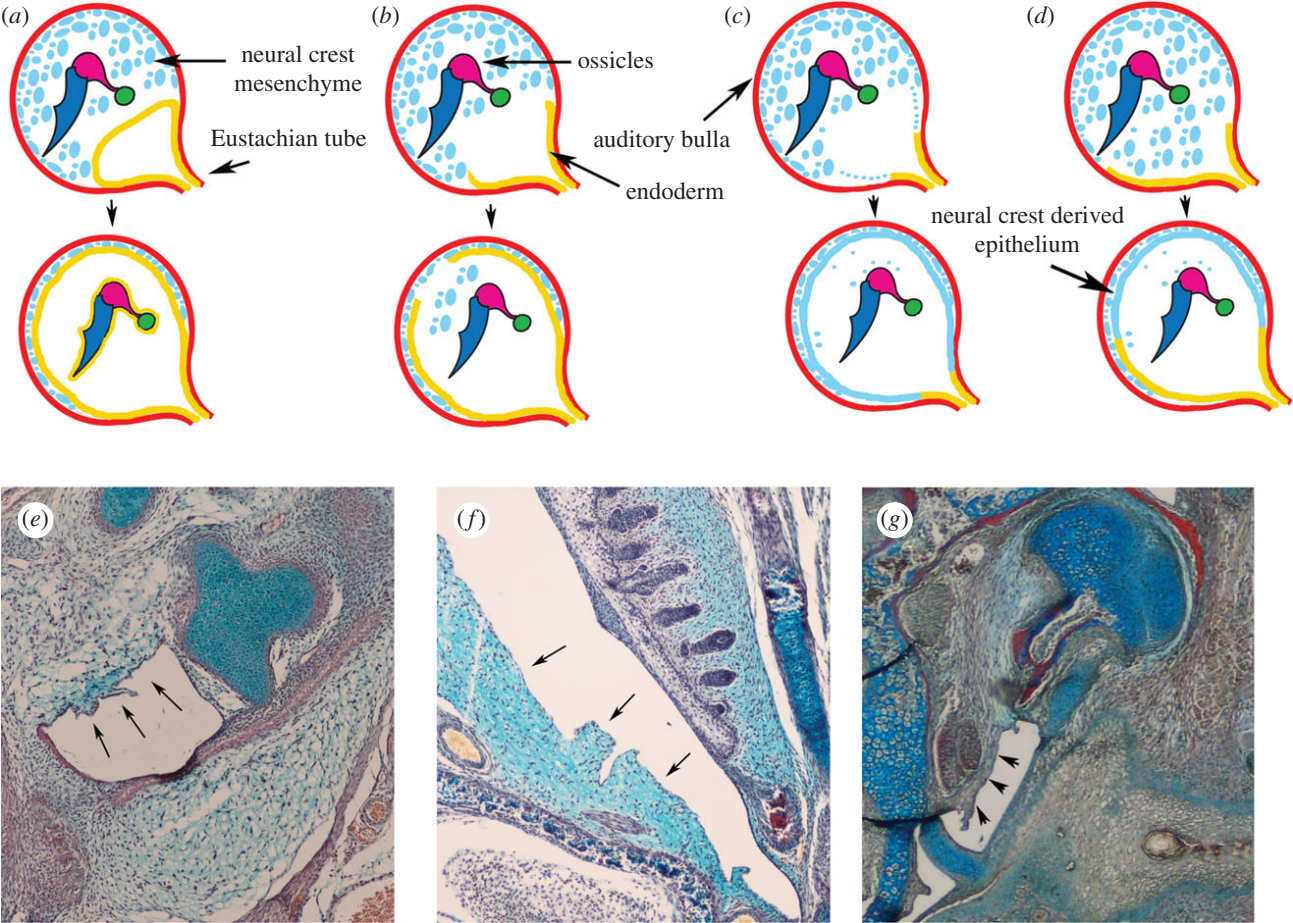
Cavitation of the ear. (*a–c*) Proposed processes of creating an air-filled space. (*a*) Invasion of the endoderm as a sheet of tissue wrapping around the ossicles. (*b*) Break of the endoderm to allow the tissue to move around the ossicles. (*c*) No invasion of the endoderm but creation of a cavity by retraction and transformation of the mesenchyme. (*d*) Process based on lineage tracing showing a dual origin of the middle ear lining incorporating some of the ideas from the previous three models. (*e–g*) Histology sections through the middle ear during retraction of the mesenchyme: (*e*) mouse E18.5; (*f*) shrew P5; (*g*) postnatal opossum. Arrows represent the mesenchyme retracting back from the forming tympanic membrane creating a cavity.

During development, the middle ear cavity has been proposed to form as an extension of the pharynx led by an outpocketing and extension of the endoderm of the first pharyngeal pouch [30]. The resulting connection between the pharynx and the middle ear is known as the pharyngotympanic tube, or the Eustachian tube in mammals (figure 1). The extension of the endoderm of the pharynx into the middle ear has been named the endodermal concept (endodermal model) and suggests the pharyngeal pouch moves into the middle ear region expanding and enveloping the middle ear structures, to result in a cavity lined completely by endoderm (figure 2*a*). In 1959, Schwarzbart proposed the mesenchymal concept, based on histological examination of a large number of human cadavers, which suggests that the endoderm ruptures and does not invade the middle ear. In this scenario, the mesenchyme retracts and forms the lining of the middle ear cavity and the mastoid air spaces [31] (figure 2*c*). Since then many scientists have published either in support of the endodermal model [32,33] or the mesenchymal model [34–36], although it is overwhelmingly the endodermal model that is described in textbooks. However, there are a number of problems with the endodermal model, principally that the middle ear cavity is not empty but contains a number of obstacles (the ossicles, blood vessels and nerves) which might prevent an epithelium expanding through as a continuous sheet. Classically, the endoderm, therefore, needs to wrap around the ossicles as it moves into the ear to create the middle ear cavity [32]. Alternatively, the endoderm might rupture during invasion of the middle ear space, to allow the epithelium to move past the various obstacles, after which the epithelium could unite to form a continuous endodermal lining (figure 2*b*).

The question of the origin of the lining of the middle ear has recently been addressed by lineage tracing in the middle ear of the mouse [37]. The endoderm, labelled with the *Sox17icre*, extends from the pharynx up along the outside of the developing otic capsule towards the base of the ossicles, forming a rudimentary middle ear cavity at embryonic day (E)15.5, as would be predicted by the endodermal model. However, after this initial extension the endoderm adjacent to the otic capsule thins and then ruptures at E17.5 in the mouse. The mechanism behind the rupture is unclear but the position of the breakdown of the endoderm right next to the otic capsule, suggests a potential role for the inner ear in controlling this step. The loss of integrity of the endoderm results in the surrounding neural crest derived mesenchyme moving in through the break in the endoderm filling the middle ear cavity of the mouse at birth. These mesenchymal cells later retract back away from the tympanic membrane and the ossicles to clear the middle ear space and reform a cavity (figure 2*e*). The cavitation process starts just after birth and is finished by post-natal day (P)14, coinciding with the onset of hearing in the mouse. The mechanism behind this retraction is unknown but appears to involve the growth of the auditory bulla, as in mice with small auditory bullae the mesenchyme often fails to clear completely (see §6) [10].

After retracting to the edges of the cavity the neural crest-derived cells, as labelled with *Wnt1cre*, then transform into an epithelium by undergoing a mesenchymal-to-epithelial transition, thereby creating a lining of dual origin [37] (figure 2*d*). In the hypotympanum at the base of the ear, near the Eustachian tube, which connects the middle ear to the pharynx, the middle ear cavity is lined by endoderm, but over the cochlea and in the attic region of the cavity around the ossicles the lining is of neural crest origin. This dual origin appears to be necessary given the three ossicle ear of mammals, which provides a particularly difficult set of obstacles to get around. A similar process of retraction of the mesenchyme can also be observed in other mammals, including the shrew and opossum, indicating a similar process across placental mammals and marsupials (figure 2*f,g*). Interestingly, this breaking of the endoderm, filling and then retraction of the mesenchyme, does not appear to occur in non-mammals where the endoderm remains intact and the middle ear space is never infilled by mesenchyme [37]. The endoderm may therefore be able to create a cavity when there is only a single ossicle to encounter. This could be tested by grafting experiments in birds and reptiles, allowing the origin of the middle ear lining throughout the cavity to be determined. The mammalian middle ear therefore not only has a different complement of ossicles, and a different mode of tympanic membrane development, but the way the cavity itself forms appears to be also unique, again supporting the view that the mammalian middle ear formed independently from that of non-mammalian middle ears. By looking at how the middle ear cavity forms, developmental biology will hopefully also be able to shed light on whether the middle ear of monotremes is homologous to that of therian mammals (marsupials and placentals), or another example of independent evolution of a tympanic ear. If the ears are homologous in all mammals, we would expect the process of cavitation, i.e. break of the endoderm, influx of the mesenchyme, retraction of the mesenchyme, transformation to an epithelium, would be conserved. Investigating how the middle ear develops in monotremes, although constrained by access to samples, will therefore be very revealing.

## 4. Atympanic middle ears

**Figure 3.**
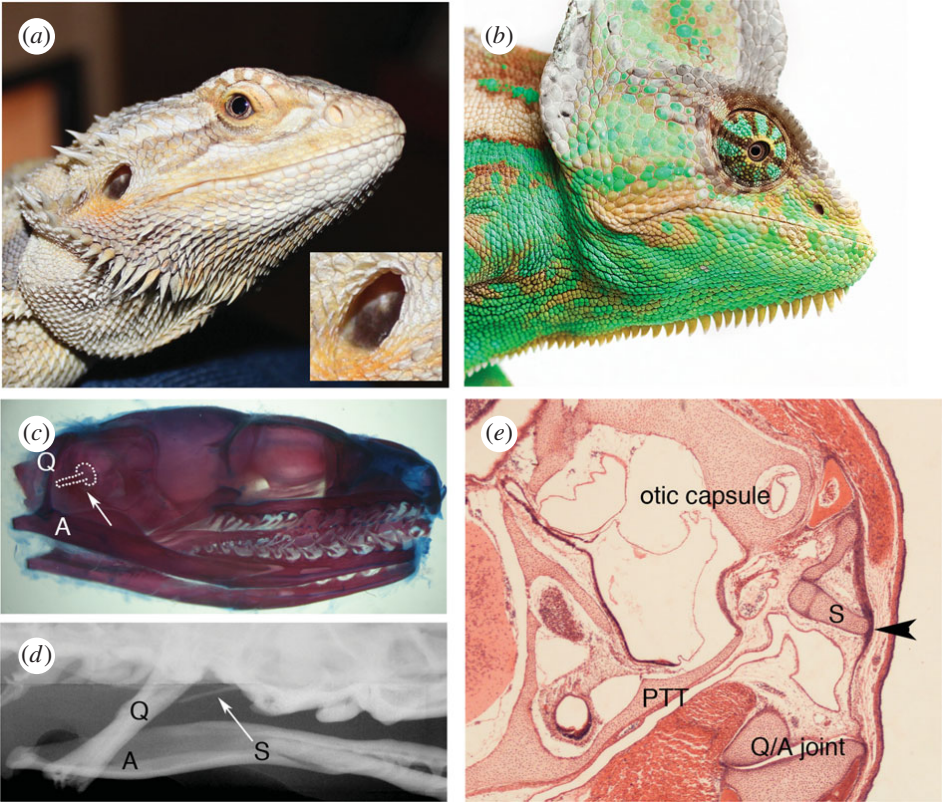
Atympanic ears. (*a*) Bearded dragon (*Pogona viviceps*) with superficial tympanic membrane. Inset shows ossicle visible through the membrane. (*b*) Chameleon (*Chamaeleo calyptratus*) showing lack of an external ear canal. (*c*) Skeletal prep of a newborn corn snake (*Elaphe guttata*), with the position of the stapes outlined (white dots). (*d*) Faxitron of adult corn snake. Arrow points to the thin stapes, which is connected to the quadrate. (*e*) *Sphenodon punctatus* approximately eight months incubation. The stapes is inserted into the otic capsule on one side and into a fibrous sheath (arrowhead) on the other. The middle ear cavity has not extended up past the stapes, which is still surrounded by mesenchyme. *Sphenodon* slides from Denby collection at KCL. Q, quadrate; A, articular; S, stapes; PTT, pharyngotympanic tube (connecting ear and pharynx).

If the tympanic membrane and cavity have evolved several times, they also appear to have been lost a number of times, at least in the diapsids (lineage of reptiles and birds). In most modern reptiles, the tympanic membrane is located at the surface and it is possible to look directly into the air-filled middle ear and see the stapes (figure 3*a*). However, in snakes and some lizards, such as the chameleon, the tympanic membrane has been completely lost (figure 3*b*). Loss of the tympanic membrane in these cases has been associated with burrowing, jaw alterations for food capture and other specializations. In the snake loss of the tympanic membrane accompanies an almost complete loss of the tympanic cavity, leaving the stapes embedded in mesenchyme and connected to the jaw apparatus, in this case the quadrate [38] (figure 3*c,d*). Snakes have been shown to be sensitive to sound-induced vibrations, rather than responding to sound pressure [39]. No tympanic membrane is also observed in *Sphenodon*, the sole surviving member of the Rhynchocephalia (the sister group of the Squamata which contains all lizards and snakes). In adult *Sphenodon*, a middle ear cavity has been described but is filled with adipose tissue [40]. As in snakes, the columella/stapes in *Sphenodon* is connected to the quadrate but in addition the columella is connected to the epihyal end of the hyoid arch [40]. In the late embryo, the endoderm of the first pharyngeal pouch does not appear to extend up into the middle ear, leaving the stapes embedded in tissue, with the lateral end pushing against a sheet of fibrous tissue instead of a tympanic membrane (figure 3*e*). The middle ear cavity, therefore, never forms in *Sphenodon*, but rather the neural crest-derived tissue surrounding the stapes would appear to differentiate into adipose. In *Sphenodon*, it has been suggested that this does not represent loss of the cavity during evolution but the retention of the original cavity-free middle ear; however, from an analysis of the fossil record it appears more likely that the *Sphenodon* ear is degenerative, i.e. these animals once possessed an air-filled cavity that has been subsequently lost (Susan Evans 2016, personal communication). In any case, on the basis of the anatomy of the middle ear and apparently unspecialized inner ear, *Sphenodon* may model the hearing ability of diapsids and their ancestors [41]. A middle ear filled with fatty tissue has also been observed in the sea turtles (Cheloniidae), associated with a reduction in the air-filled space [42]. In comparison to loss of the tympanic cavity and ear drum in reptiles, the lack of a tympanic cavity in Gymnophiona (Caecilians) and Caudata (salamander and newts) is likely to be retention of a primitive character [20,43].

## 5. Connecting the ear to the pharynx

**Figure 4.**
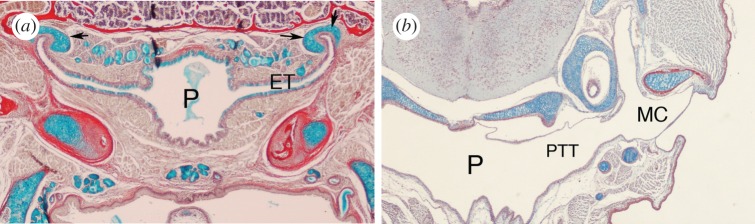
Connection to the pharynx. (*a*) Frontal section through the Eustachian tube (ET) in a mouse P22. The narrow ET connects the middle ear (out of plane of section) to the naso-pharynx (P). The tube is supported by cartilage (arrows) and is lined with mucin-producing cells (stained blue). (*b*) Frontal section through a gecko embryo. The middle ear cavity (MC) is connected to the pharynx (P) via a wide tube, the pharyngotympanic tube (PTT).

The connection of the middle ear to the pharynx, formed during development through the extension of the first pharyngeal pouch, is retained in the ears of anurans, birds, reptiles and mammals as the pharyngotympanic tube. This connection allows pressure in the middle ear to be equalized by air moving between the pharynx and the ear. The structure of this tube is very different in mammals compared to most reptiles, with mammals having a narrow tube, known as the Eustachian tube, while reptiles have a wide-open connection between the pharynx and ear (figures 1 and 4*a,b*).

The narrow tube found in mammals is thought to have been constricted by growth of the brain, along with changes in head anatomy triggered by development of the secondary palate [44]. Together these two anatomical changes potentially led to the narrow tube observed in modern mammals; however, as avians have expanded brains and secondary palate-like structures it is unclear whether such a mechanical argument is valid. In contrast to this narrow arrangement, the platypus has been shown to have very wide Eustachian tubes, so that the middle ear is freely connected to the pharynx [45]. This arrangement is not observed in echidna (spiny anteaters) so it is not a general feature of monotremes. The mammalian Eustachian tube is supported by cartilage and can be opened and closed by muscles, such as the first arch derived tensor veli palatini and the fourth arch-derived levator veli palatini muscles [46]. The tensor veli palatini is innervated by the pterygoid nerve and has been proposed to be homologous to one of the pterygoid muscles in non-mammals, although homology to the adductor mandibularis has also been suggested [47,48]. The Eustachian tube is usually found in a collapsed form and is opened in association with actions such as swallowing, allowing pressure in the middle ear to be equalized with that of the pharynx. Defects in the development of these muscles, as observed in *Tbx1* mutant mice, lead to problems of clearance of the middle ear resulting in ear infection (otitis media) [49]. The tube is highly ciliated to waft debris out of the ear. Cilia are also associated with the lower part of the middle ear cavity in mammals, in the part of the ear lined by endoderm, acting to funnel debris towards the mouth of the Eustachian tube. By contrast, the neural crest lined part of the mammalian cavity does not form these motile cilia [37]. Interestingly, cilia are not associated with the middle ear mucosa in birds, although cilia are associated with the pharyngotympanic tube [50]. The appearance of cilia in mammalian middle ears appears essential due to the narrowness of the Eustachian tube, but also its position, as the tube forms at the side rather than the base of the middle ear cavity [51]. A ciliated epithelium, therefore, appears to have evolved in mammalian ears as a later adaptation to changes in the connection of the ear and pharynx, with cilia extending from the ciliated pharyngotympanic tube into the middle ear. If cilia are defective, as in *Spag6* mutant mice, fluid and mucous accumulate in the middle ear leading to inflammatory diseases of the middle ear (otitis media) [52]. Otitis media is also associated with patients with primary ciliary dyskinesia [53,54].

In most lizards, the middle ear falls seamlessly into the pharynx with no clear cut off between the middle ear cavity and mouth, and therefore gravity alone would clear the ear (figure 4*b*). In turtles, however, a narrow tube, similar to the arrangement in mammals, closes off the connection between the pharynx and the middle ear. In this case, the arrangement has been linked to the ability to hear sound underwater [42].

In crocodilians, there is a complex network of airspaces that actually connect the ears on either side of the head by means of a bony canal that runs over the brain case [55]. A similar connection between the two ears occurs in birds. In this case, the pharyngotympanic tube runs in an ossified canal along the parasphenoid and basitemporal plate, with the two tubes from either side of the head fusing before opening into the pharynx [56]. The connection between the two ears in birds and crocodiles allows for an internal acoustic pathway and coupling of the ears. This allows for a directional response from the tympanic membrane improving sound localization (discussed in [57]). A similar coupling of the ears can be observed in frogs and lizards, but in this case the ears are coupled through the pharynx and the wide-open pharyngotympanic tube. In most mammals, with their narrow Eustachian tubes such coupling is not possible and therefore mammals rely on neural computation to interpret sound information arriving separately on either side of the head [58]. There are, however, some mammals where the middle ear cavities are linked up and where intercommunication is therefore possible, such as in some talpids (moles) and the golden mole [57]. How the connection between ears in these species is controlled during development is unknown but would involve aeration of the basicranial bones, perhaps in a similar process as observed in the formation of the mastoid air spaces in the temporal bone of many mammals.

Motile cilia in the middle ear and a muscle network to open and close the connection between the pharynx and the ear therefore appear to be additional mammalian novelties. The middle ear has not been studied well in monotremes, due to the difficulty of obtaining specimens, but a ciliated middle ear may not be necessary in the platypus due to the relatively open connection of the ear with the pharynx.

## 6. Enclosing the middle ear

In most therian mammals (marsupials and placentals), the middle ear is encased by a bony structure known as the auditory bulla that protects the middle ear space, and forms the floor of the middle ear cavity. Such a structure has not been observed in monotremes [59], so some middle ear features are not common to all mammals. The bulla protects the middle ear tissues and is thought to have developed fairly early on in therian evolution. The bulla may therefore represent a synapomorphy (shared derived character) to define therians. However, the composition of the bulla in mammals is very variable with contribution from the tympanic ring (ectotympanic), the homologue of the reptilian angular, the squamosal, petrosal, entotympanic, alisphenoid and basisphenoid [59]. Interestingly, some mammals have incomplete auditory bulla and therefore have only partially enclosed ears. This is observed in opossums, shrews and some talpid moles [57]. In the opossum, the tympanic ring is loosely suspended. In most mammals, in contrast, the tympanic ring is firmly synostosed to the skull (squamosal and petrosal bones), which in humans form the compound temporal bone. In the mouse, a recognizable bulla can be observed postnatally at P6, with the most notable growth occurring between P9 and P14, the time-point at which most of the neural crest-derived mesenchyme retracts back to leave the air-filled space [10,37]. The development of the auditory bulla has been associated with the process of cavitation in the mouse with *Tcof1* mouse mutants (model of Treacher–Collins syndrome) with small bulla being associated with poor clearance of the mesenchyme from the ear and high levels of otitis media [10]. A similar link between cavitation failure and small bulla size is observed in hypophysectomized Long-Evan rats and Snell dwarf mice that have combined pituitary hormone deficiency [60,61] and in *Eya1* (model of branchio-oto-renal syndrome) and *Eya4* mice [62,63]. Deafness, retained mesenchyme and defects in the auditory bulla have also been observed in mice with mutations in thyroid hormone receptor [64], indicating that thyroid hormone signalling is important for mesenchymal clearance. Interestingly, injections of anterior pituitary extract into strain-dependent deaf rats has been reported to lead to a reduction in retained mesenchyme and a rescue of hearing [65]: whether these rats also had rescued bulla defects, however, was not reported. It is therefore unclear whether the failure in growth of the bulla directly leads to a failure in complete cavitation (or vice versa), i.e. is the primary cause, or whether the two processes are both secondary to an earlier defect.

## 7. The three ossicle ear

So why did mammals evolve a three ossicle middle ear rather than a single ossicle ear? This appears to revolve around changes to the mammalian jaw joint, which started with changes to the teeth [66,67]. Mammals are able to chew and often have excellent occlusion between their upper and lower teeth allowing grinding. This is achieved by the jaws being able to move sideways, in addition to up and down, with the teeth on the upper and lower jaws fitting together when the mouth is closed. By contrast, toothed non-mammals generally have jaw movement limited to up and down, preventing chewing, and the teeth are not in occlusion. Chewing is possible due to the development of a novel mammalian jaw joint between the squamosal (upper jaw) and dentary (lower jaw) known as the temporomandibular joint in humans. From the fossil record, the basal mammaliforms, such as *Morganucodon*, showed occlusion between their teeth and the presence of a double jaw joint [68]. This jaw joint was made up of the normal reptilian style jaw joint, between the articular (lower jaw) and the palatoquadrate (upper jaw) plus a new joint between an expanded dentary bone in the lower jaw and the squamosal bone in the upper jaw. This new joint, forming from dermal (membranous) ossification, appeared to provide a stabilizing force allowing more movement of the lower jaw and preventing dislocation. Shearing occlusion between complicated postcanine teeth, however, was only observed after the advent of this second jaw articulation [69]. It is proposed that the advent of the new joint then freed up the existing joint (articular, quadrate) to start playing a role in hearing. It is often assumed that the three ossicle mammalian middle ear is superior to the single ossicle tympanic middle ears of other animals, although there is very little evidence to support this and in some ways the mammalian ear is more derived, for example, in the relationship of the stapes and incus (hyomandibular and quadrate) [70].

That the articular and quadrate part of the palatoquadrate are homologous to the malleus and incus in the mammalian middle ear was first proposed by Reichert [71] and extended by Gaupp [18] based on comparative anatomy. Developmental biology has been able to shed light on this proposed homology by showing that during embryonic development the malleus and incus develop connected to Meckel's cartilage with a joint developing between them to separate them in an identical process to that observed for the articular and quadrate in non-mammalian jawed vertebrates [21,72]. The expression of key joint genes, such as *Bapx1*, are also conserved between the jaw joint and ear joint in non-mammals and mammals, respectively [21,72–74]. Fate mapping studies have shown that the quadrate and articular of the chick are derived from first arch neural crest, while the retroarticular process that extends from the articular is second arch derived [3,75]. In keeping with the proposed homology, the malleus and incus are also first arch derived except for a small structure known as the orbicular apophysis on the malleus which is second arch derived [11]. The retroarticular process of the chick therefore appears homologous to the orbicular apophysis of the mouse, based on arch derivation and respective position on the articular and malleus. Such fate mapping experiments rule out the suggested homology of the retroarticular process with the manubrium, an extension of the malleus that inserts on the ear drum, as the manubrium is first arch derived [12,19]. The manubrium of the malleus therefore represents a neomorphic structure [76].

In non-mammalian amniotes, the articular and quadrate form as the most proximal part of Meckel's cartilage, with the articular remaining attached to Meckel's cartilage, which is persistent throughout the animal's life. In mammals, however, for the malleus and incus to take part in hearing they need to separate from Meckel's and the rest of the lower jaw. In the mouse, this process starts soon after birth with the part of Meckel's next to the ossicles transforming into the sphenomandibular ligament [74]. This process is particularly interesting to follow in marsupials. Owing to their short gestation, marsupials are born before the formation of the dentary–squamosal joint [77,78]. At birth, therefore, the point of articulation between the upper and lower jaw is centred around the cartilaginous middle ear bones which are still attached to Meckel's cartilage [79]. The ear bones at birth are large and appear to buttress the jaw against the skull; however, no synovial joint is yet evident between the malleus and incus and the ear ossicles probably do not function as a true jaw joint in the neonate [80]. Suckling may, therefore, be possible due to flexibility of Meckel's as a cartilaginous rod. Despite this the move from a function in the jaw to a function in the ear can be followed during neonate development as the mammalian jaw joint forms and the relative function of the ossicles shifts. By 26 days after birth the middle ear cavity has formed an air-filled space in *Monodelphis domesticus* and the animals are able to hear by 28–30 days, indicating the shift to an ear function has happened by this time-point [81].

The joint between the malleus and incus in mammals still appears to play an important role in patterning the upper and lower jaws, despite it no longer being the functional site of articulation between these structures in the adult. In the *Hoxa2* mutant, mirror image duplications are observed centred around the malleus and incus, and in endothelin knockouts and *Dlx5/6* double mutants the transformation of upper and lower jaw structures is based around the jaw joint [24,82]. This has led to the idea that the primary middle ear joint (the hinge) is a signalling centre which, working with the cells at the end of the jaw (the caps), provides positional information along the jaw in all jawed vertebrates, irrespective of subsequent modifications [83].

## 8. The novel jaw joint

As mentioned in §7, it is thought that the novel jaw joint stabilized the jaw, preventing dislocation during chewing. In extant mammals, this joint is created between the squamosal and dentary, two dermal bones that in reptiles are placed far apart in the jaw. In the fossil record, there are examples of non-mammalian synapsids, such as *Ictidopsis* [84,85] with upwardly extending dentaries that might represent a step towards this novel jaw articulation, while some mammal-like synapsid groups, tritheledontids and brasilodontids, have a ridge on the dentary that contacts the squamosal, forming a hinge-like structure [86].

**Figure 5.**
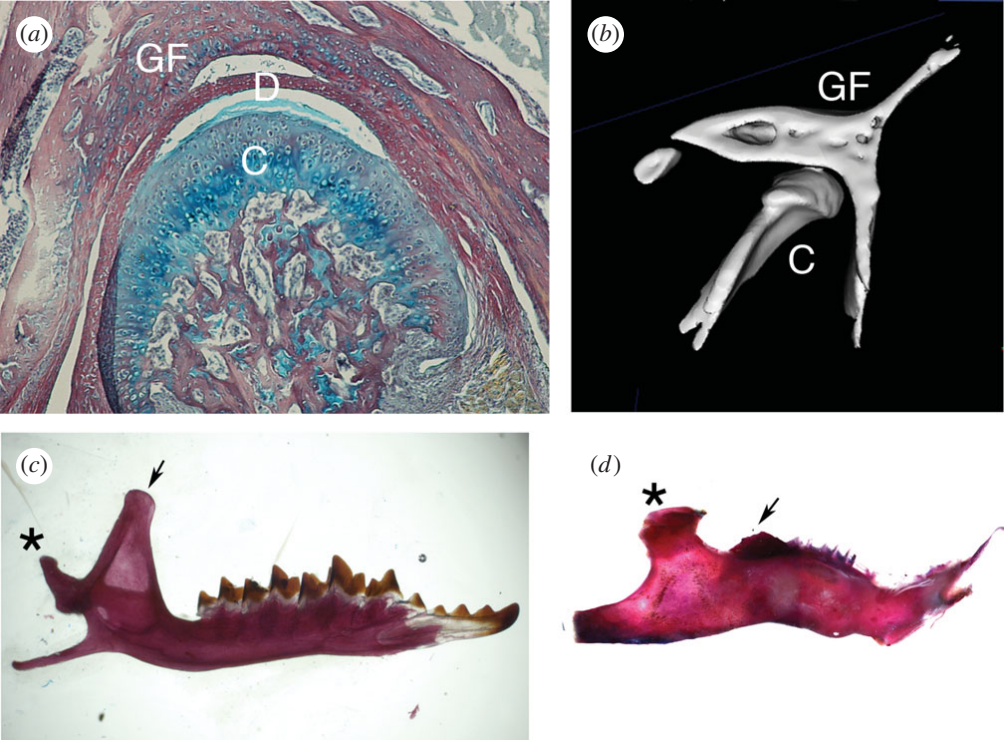
The novel mammalian jaw joint. (*a*) Histology section through a weaning mouse showing the condylar cartilage (C), disc (D) and glenoid fossa (GF). (*b*) MicroCT of the condyle sitting in the glenoid fossa in an adult mouse. (*c*) Skeletal prep of adult shrew dentary with large coronoid. (*d*) Skeletal prep of adult guinea pig dentary with almost complete loss of the coronoid. Asterisks indicate condyle; arrows point to coronoids.

In order for a functional joint to form between the dentary and squamosal bones, a cartilage cap develops on the dentary, known as the condyle (figure 5*a*). It is the condylar process of the dentary that articulates with the glenoid fossa of the squamosal bone. This condylar cartilage is a secondary cartilage, forming after the formation of dermal bone. It is unclear whether the cartilage develops as a sesamoid, i.e. an independent condensation, or from the periosteum of the neighbouring dermal bone. Evidence in rats and mice has suggested the condylar process forms as a sesamoid [87–89]; however, a recent immunohistological analysis in rats, mouse and human developing jaws suggests that the developing condylar process forms with the dentary and is derived from the periosteum [90]. In keeping with this, no condylar process forms in the absence of the dentary in *Runx2* mutant mice; however, condensed mesenchyme in the regions where the secondary cartilages would normally form was observed [91]. *Bmp2* was able to rescue the secondary cartilage in *Runx2* mice, suggesting that the cartilages can form in the absence of a periosteum [92]. Given the conflicting evidence, it will be important to follow up these findings using lineage tracing of the periosteum, to confirm that the condylar cartilage does indeed form from this tissue. If the condylar process develops from the periosteum this may involve a similar mechanisms as observed in birds, where the secondary cartilages have been shown to be derived from the periosteum [93]. Avian secondary cartilages, however, differ in their development in that they require mechanical stimulation for their induction while mammalian secondary cartilages appear not to as they can form in culture [87,93–95]. It appears likely that secondary cartilages evolved independently in birds and mammals as amphibians and reptiles do not appear to have secondary cartilage [96].

During development the top layer of the forming condyle lifts off from the cartilage layers below and forms the disc that sits between the glenoid flossa and condylar process and forms an integral part of the joint. Formation of this disc has been shown to involve Indian hedgehog signalling at at least two time points, first to allow the disc to form, and then for it to separate from the condyle [97,98]. BMP signalling has also recently been shown to be essential for murine jaw joint formation, with both gain and loss of function experiments generating defective joints [99]. BMP appears to act upstream of Hedgehog signalling, with loss of the BMP receptor *Bmpr1a* in the neural crest leading to downregulation of Indian hedgehog in the condylar process and a failure in disc formation [99].

The condyle fits perfectly in the glenoid fossa, which forms a socket around the developing cartilage and disc (figure 5*b*). In the absence of the condylar, the shape of the glenoid fossa is disrupted with loss of the lateral wall, suggesting important interactions between these two sides of the jaw [100]. During later development, the condylar process acts as a growth centre with growth of the dentary occurring as cartilaginous cells within the condyle start to undergo hypertrophy leading to endochondral ossification. Interestingly, it has recently been shown by lineage labelling that the majority of cartilage cells of the condyle directly transform into bone cells during this process, rather than the previous view that the cells died and were replaced by invading osteoblasts [101]. The cells of this secondary cartilage therefore physically take part in the jaw bone, regulating its size and shape.

## 9. Modularity of the mammalian dentary

The dentary bone in mammals replaces a number of bones (angular, surangular, splenial and prearticular) that serve this role in non-mammalian jawed vertebrates. As mentioned in §2, the post-dentary jaw bones, such as the angular and prearticular, have ended up with new roles in the ear as support for the malleus and the tympanic membrane. The post-dentary surangular is also associated with the mammalian ear, and is thought to be homologous to the accessory malleus, a small bone lying above the anterior process of the malleus, observed in a few mammals but not the mouse [102]. The dentary of mammals is a modular structure with different modules taking on different roles of the lower jaw, for example, articulation site (condyle) and muscle attachment sites for jaw opening and closing (angular process and coronoid process) [103]. This modularity is emphasized in mouse mutants where specific parts of the dentary are lost. For example, the coronoid process is lost in *Pax9* mutant mice and in *Tbx1* mutant mice [104,105], while the angular process in lost in *Tgfb2* mutants [106]. This independent regulation of different parts of the dentary appears to allow one module to change without deleteriously affecting the other parts. This is dramatically observed in many herbivores where the coronoid is almost completely lost during development without affecting the articulation function of the jaw, in contrast to carnivores/insectivores which have large coronoids with a large muscle attachment and a fierce bite [103] (figure 5*c,d*). The initiation of the coronoid involves *Pax9*, while the growth of the coronoid after initiation is influenced by muscle attachment, which triggers expression of *Sox9*. Thus herbivores, such as the guinea pig, have less fewer muscles attaching to the coronoid during development compared with omnivores such as the mouse, resulting in reduced expression of *Sox9* and reduced relative growth of this skeletal element [107].

In conclusion, a tympanic middle ear has evolved independently numerous times, incorporating one ossicle in birds and reptiles, but three ossicles in mammals. Developmental biology has played an important role in unmasking the different mechanisms by which the ear forms in mammals and sauropsids, highlighting the different possible ways to make an external ear canal, an ear drum, an air-filled space, a Eustachian tube and a bulla. The use of time and tissue-specific transgenic mice, new lineage tracing techniques and an increased access to non-model organisms such as the opossum, means that many of the open questions surrounding the evolution of the middle ear are starting to be answered, and we have a really exciting period of research ahead.

## References

[1] Lindquist ED, Hetherington TE, Volman SF 1998 Biomechanical and neurophysiological studies on audition in eared and earless harlequin frogs (*Atelopus*). J. Comp. Physiol. A 183, 265–271. (10.1007/s003590050254) 9693994

[2] Boistel R, Aubin T, Cloetens P, Peyrin F, Scotti T, Herzog P, Gerlach J, Pollet N, Aubry J-F 2013 How minute sooglossid frogs hear without a middle ear. Proc. Natl Acad. Sci. USA 110, 15 360–15 364. (10.1073/pnas.1302218110) PMC3780892 24003145

[3] Kontges G, Lumsden A 1996 Rhombencephalic neural crest segmentation is preserved throughout craniofacial ontogeny. Development 122, 3229–3242. 8898235 10.1242/dev.122.10.3229

[4] Noden DM 1986 Origins and patterning of craniofacial mesenchymal tissues. J. Craniofac. Genet. Dev. Biol. Suppl. 2, 15–31. 3491109

[5] Zou Y, Mak SS, Liu HZ, Han DY, Zhuang HX, Yang SM, Ladher RK 2012 Induction of the chick columella and its integration with the inner ear. Dev. Dyn. 241, 1104–1110. (10.1002/dvdy.23788) 22473893

[6] Toerien MJ 1963 Experimental studies on the origin of the cartilage of the auditory capsule and columella in *Ambystoma*. J. Embryol. Exp. Morphol. 11, 459–473. 14061953

[7] Toerien MJ 1965 Experimental studies on the columella-capsular interrelationship in the turtle *Chelydra serpentina*. J. Embryol. Exp. Morphol. 14, 265–272. 5861722

[8] Reagan FP 1917 The role of the auditory sensory epithelium in the formation of the stapedial plate. J. Exp. Zool. 23, 85–108. (10.1002/jez.1400230104)

[9] Thompson H, Ohazama A, Sharpe PT, Tucker AS 2012 The origin of the stapes and relationship to the otic capsule and oval window. Dev. Dyn. 241, 1396–1404. (10.1002/dvdy.23831) 22778034

[10] Richter CA, Amin S, Linden J, Dixon J, Dixon MJ, Tucker AS 2010 Defects in middle ear cavitation cause conductive hearing loss in the *Tcof1* mutant mouse. Hum. Mol. Genet. 19, 1551–1560. (10.1093/hmg/ddq028) 20106873

[11] O'Gorman S 2005 Second branchial arch lineages of the middle ear of wild-type and *Hoxa2* mutant mice. Dev. Dyn. 234, 124–131. (10.1002/dvdy.20402) 15861402

[12] Shute CC 1956 The evolution of the mammalian eardrum and tympanic cavity. J. Anat. 90, 261–281. 13319213 PMC1244848

[13] Westoll TS 1945 The mammalian middle ear. Nature 155, 114–115. (10.1038/155114a0)

[14] Clack JA 2002 Patterns and processes in the early evolution of the tetrapod ear. J. Neurobiol. 53, 251–264. (10.1002/neu.10129) 12382279

[15] Manley GA, Clack JA 2004 An outline of the evolution of vertebrate hearing organs. In Evolution of the vertebrate auditory system (eds Manley GA, Popper A, Fay RR), pp. 1–26. New York, NY: Springer.

[16] Rich TH, Hopson JA, Musser AM, Flannery TF, Vickers-Rich P 2005 Independent origins of middle ear bones in monotremes and therians. Science 307, 910–914. (10.1126/science.1105717) 15705848

[17] Luo ZX 2007 Transformation and diversification in early mammal evolution. Nature 450, 1011–1019. (10.1038/nature06277) 18075580

[18] Gaupp E 1913 Die Reichertsche Theorie (Hammer-, Ambos- und Kiefertrage). *Archiv Anat. Phyriol. Abt. Anat. Entwicklungsgesch. Suppl.-Band*, 1–416.

[19] Goodrich E 1930 Studies on the structure and development of vertebrates. London, UK: Macmillan.

[20] Lombard RE, Bolt JR 1979 Evolution of the tetrapod ear: an analysis and reinterpretation. Biol. J. Linn. Soc. 11, 19–76. (10.1111/j.1095-8312.1979.tb00027.x)

[21] Tucker AS, Watson RP, Lettice LA, Yamada G, Hill RE 2004 *Bapx1* regulates patterning in the middle ear: altered regulatory role in the transition from the proximal jaw during vertebrate evolution. Development 131, 1235–1245. (10.1242/dev.01017) 14973294

[22] Kitazawa T et al. 2015 Developmental genetic bases behind the independent origin of the tympanic membrane in mammals and diapsids. Nat. Commun. 6, 6853 (10.1038/ncomms7853) 25902370 PMC4423235

[23] Presley R 1984 Lizards, mammals and the primitive tetrapod tympanic membrane. In The structure, development and evolution of reptiles (ed. Ferguson MWJ), pp. 127–152. London, UK: Academic Press.

[24] Sato T et al. 2008 An endothelin-1 switch specifies maxillomandibular identity. Proc. Natl Acad. Sci. USA 105, 18 806–18 811. (10.1073/pnas.0807345105) PMC2596216 19017795

[25] Michaels L, Soucek S 1989 Development of the stratified squamous epithelium of the human tympanic membrane and external canal: the origin of auditory epithelial migration. Am. J. Anat. 184, 334–344. (10.1002/aja.1001840408) 2756906

[26] Nishizaki K, Anniko M, Orita Y, Karita K, Masuda Y, Yoshino T 1998 Programmed cell death in the developing epithelium of the mouse inner ear. Acta Otolaryngol. 118, 96–100. (10.1080/00016489850155206) 9504171

[27] Grevellec A, Tucker AS 2010 The pharyngeal pouches and clefts: development, evolution, structure and derivatives. Semin. Cell Dev. Biol. 21, 325–332. (10.1016/j.semcdb.2010.01.022) 20144910

[28] Minoux M, Kratochwil CF, Ducret S, Amin S, Kitazawa T, Kurihara H, Bobola N, Vilain N, Rijli FM 2013 Mouse *Hoxa2* mutations provide a model for microtia and auricle duplication. Development 140, 4386–4397. (10.1242/dev.098046) 24067355

[29] Rijli FM, Mark M, Lakkaraju S, Dierich A, Dolle P, Chambon P 1993 A homeotic transformation is generated in the rostral branchial region of the head by disruption of *Hoxa-2*, which acts as a selector gene. Cell 75, 1333–1349. (10.1016/0092-8674(93)90620-6) 7903601

[30] Wittmaack K 1918 Uber die Normale und die Pathologische Pneumatisation des Schlaefenbeines. Jena, Germany: Gustav Fischer.

[31] Schwarzbart A 1959 The pneumatization of the temporal bone: a new concept. J. Laryngol. Otol. 73, 45–47. (10.1017/S0022215100054906) 13621065

[32] Proctor B 1964 The development of the middle ear spaces and their surgical significance. J. Laryngol. Otol. 78, 631–648. (10.1017/S002221510006254X) 14193231

[33] Hilding DA, Szachowicz E, Larsen SA 1980 Development of the epithelium of the middle ear. Electron microscopic study of fine structure, including junctional complexes and basal lamina. Am. J. Otolaryngol. 1, 97–108. (10.1016/S0196-0709(80)80002-0) 7446840

[34] Buch NH, Jorgensen MB 1964 Eustachian tube and middle ear. Embryology and pathology. Arch Otolaryngol. 79, 472–480. (10.1001/archotol.1964.00750030483007) 14120670

[35] Foley JF, Guggenheim P, Clements LP 1965 Tissue culture of rat tympanal mesenchyme. Acta Otolaryngol. 60, 531–538. (10.3109/00016486509127036) 5882310

[36] Marovitz WF, Porubsky ES 1971 The embryological development of the middle ear space—a new concept. Ann. Otol. Rhinol. Laryngol. 80, 384–389. (10.1177/000348947108000313) 5578785

[37] Thompson H, Tucker AS 2013 Dual origin of the epithelium of the mammalian middle ear. Science 339, 1453–1456. (10.1126/science.1232862) 23520114

[38] Wever EG 1978 The reptile ear. Princeton, NJ: Princeton University Press.

[39] Christensen CB, Christensen-Dalsgaard J, Brandt C, Madsen PT 2012 Hearing with an atympanic ear: good vibration and poor sound-pressure detection in the royal python, *Python regius*. J. Exp. Biol. 215, 331–342. (10.1242/jeb.062539) 22189777

[40] Gans C, Wever EG 1976 Ear and hearing in *Sphenodon punctatus*. Proc. Natl Acad. Sci. USA 73, 4244–4246. (10.1073/pnas.73.11.4244) 1069313 PMC431402

[41] Clack JA 1997 The evolution of tetrapod ears and the fossil record. Brain Behav. Evol. 50, 198–212. (10.1159/000113334) 9310195

[42] Willis KL, Christensen-Dalsgaard J, Ketten DR, Carr CE 2013 Middle ear cavity morphology is consistent with an aquatic origin for testudines. PLoS ONE 8, e54086 (10.1371/journal.pone.0054086) 23342082 PMC3544720

[43] Christensen CB, Lauridsen H, Christensen-Dalsgaard J, Pedersen M, Madsen PT 2015 Better than fish on land? Hearing across metamorphosis in salamanders. Proc. R. Soc. B 282, 20141943 (10.1098/rspb.2014.1943) PMC4344139 25652830

[44] Manley GA 2010 An evolutionary perspective on middle ears. Hear. Res. 263, 3–8. (10.1016/j.heares.2009.09.004) 19786082

[45] Denker A 1901 Zur Anatomie des Gehörorgans der Monotremata Denkschriften der Medicinisch-naturwissenschaftlichen. Gesellschaft Jena 6, 635–662.

[46] Ishijima K, Sando I, Balaban CD, Miura M, Takasaki K 2002 Functional anatomy of levator veli palatini muscle and tensor veli palatini muscle in association with Eustachian tube cartilage. Ann. Otol. Rhinol. Laryngol. 111, 530–536. (10.1177/000348940211100609) 12090709

[47] Diogo R, Abdala V, Lonergan N, Wood BA 2008 From fish to modern humans—comparative anatomy, homologies and evolution of the head and neck musculature. J. Anat. 213, 391–424. (10.1111/j.1469-7580.2008.00953.x) 18657257 PMC2644766

[48] Westoll TS 1943 The hyomandibular of Eusthenopteron and the tetrapod middle ear. Proc. R. Soc. Lond. B 131, 393–414. (10.1098/rspb.1943.0014)

[49] Fuchs JC, Linden JF, Baldini A, Tucker AS 2015 A defect in early myogenesis causes otitis media in two mouse models of 22q11.2 deletion syndrome. Hum. Mol. Genet. 24, 1869–1882. (10.1093/hmg/ddu604) 25452432 PMC4355021

[50] Giannessi F, Giambelluca MA, Scavuzzo MC, Fattori B, Ruffoli R 2003 Ultrastructural and ultracytochemical study of the middle ear epithelium in the chicken, *Gallus gallus domesticus*. J. Morphol. 256, 371–378. (10.1002/jmor.10101) 12655618

[51] Park K, Lim DJ 1992 Luminal development of the Eustachian tube and middle ear: murine model. Yonsei Med. J. 33, 159–167. (10.3349/ymj.1992.33.2.159) 1413893

[52] Li X, Xu L, Li J, Li B, Bai X, Strauss JF 3rd, Zhang Z, Wang H 2014 Otitis media in sperm-associated antigen 6 (*Spag6*)-deficient mice. PLoS ONE 9, e112879 (10.1371/journal.pone.0112879) 25393619 PMC4231073

[53] Leigh MW, Pittman JE, Carson JL, Ferkol TW, Dell SD, Davis SD 2009 Clinical and genetic aspects of primary ciliary dyskinesia/Kartagener syndrome. Genet. Med. 11, 473–487. (10.1097/GIM.0b013e3181a53562) 19606528 PMC3739704

[54] Majithia A, Fong J, Hariri M, Harcourt J 2005 Hearing outcomes in children with primary ciliary dyskinesia—a longitudinal study. Int. J. Pediatr. Otorhinolaryngol. 69, 1061–1064. (10.1016/j.ijporl.2005.02.013) 16005347

[55] Dufeau DL, Witmer LM 2015 Ontogeny of the middle-ear air-sinus system in *Alligator mississippiensis* (Archosauria: Crocodylia). PLoS ONE 10, e0137060 (10.1371/journal.pone.0137060) 26398659 PMC4580574

[56] Vorster W, Starck JM 2003 Anatomy of the middle ear of the Japanese crane *Grus japonensis* (Gruidae: Aves). J. Morphol. 257, 260–269. (10.1002/jmor.10075) 12833369

[57] Mason MJ 2016 Internally coupled ears in living mammals. Biol. Cybern. 110, 345–358. 26794500 10.1007/s00422-015-0675-1 PMC5107206

[58] Christensen-Dalsgaard J, Carr CE 2008 Evolution of a sensory novelty: tympanic ears and the associated neural processing. Brain Res. Bull. 75, 365–370. (10.1016/j.brainresbull.2007.10.044) 18331899 PMC3269633

[59] Novacek M 1993 Patterns of diversity in the mammalian skull. In The skull, vol. 2 (eds Hanken B, Hall BK), pp. 438–524. Chicago, IL: University of Chicago Press.

[60] Marovitz WF, Berryhill BH, Rowley TR 1968 Effects of early hypophysectomy on the development of the ossicular chain of rats. Laryngoscope 78, 309–315. (10.1288/00005537-196803000-00001) 4171061

[61] Marovitz WF, Berryhill BH, Peterson RR 1968 Disruptions of bony labyrinth, ossicular chain and tympanic bullae in dwarf mice. Laryngoscope 78, 863–872. (10.1288/00005537-196805000-00018) 5651419

[62] Depreux FF et al. 2008 *Eya4*-deficient mice are a model for heritable otitis media. J. Clin. Invest. 118, 651–658. 18219393 10.1172/JCI32899 PMC2213371

[63] Xu PX, Adams J, Peters H, Brown MC, Heaney S, Maas R 1999 *Eya1*-deficient mice lack ears and kidneys and show abnormal apoptosis of organ primordia. Nat. Genet. 23, 113–117. (10.1038/12722) 10471511

[64] Cordas EA, Ng L, Hernandez A, Kaneshige M, Cheng SY, Forrest D 2012 Thyroid hormone receptors control developmental maturation of the middle ear and the size of the ossicular bones. Endocrinology 153, 1548–1560. (10.1210/en.2011-1834) 22253431 PMC3281545

[65] Guggemheim L 1943 Therapy of deafness. Report of cases. Laryngoscope 53, 503–518.

[66] Crompton AW 1963 On the lower jaw of *Diarthrognathus* and the origin of the mammalian lower jaw. Proc. Zool. Soc. London. 140, 697–753.

[67] Crompton AW, Hylander WL 1986 Changes in mandibular function following the acquisition of a dentary-squamosal joint. In The ecology and biology of mammal-like reptiles (eds Hotton N, MacLean PD, Roth JJ, Roth EC), pp. 263–282. Washington, DC: Smithsonian Institute Press.

[68] Kermack KA, Mussett AF, Rigney HW 1981 The skull of *Morganucodon*. Zool. J. Linn. Soc. 71, 1–158. (10.1111/j.1096-3642.1981.tb01127.x)

[69] Maier W 1978 Die Evolution der tribosphenischen Säugetiermolaren. Sonderb. Naturwiss. Ver. Hamburg 3, 41–60.

[70] Takechi M, Kuratani S 2010 History of studies on mammalian middle ear evolution: a comparative morphological and developmental biology perspective. J. Exp. Zool. B Mol. Dev. Evol. 314, 417–433. (10.1002/jez.b.21347) 20700887

[71] Reichert K 1837 Uber die Visceralbogen derWirbeltiere im Allgemeinen und deren Metamorphosen bei den Vögeln und Säugetieren. Archiv. Anat. Physiol. Wissensch. Medizin. 120–222.

[72] Wilson J, Tucker AS 2004 Fgf and Bmp signals repress the expression of *Bapx1* in the mandibular mesenchyme and control the position of the developing jaw joint. Dev. Biol. 266, 138–150. (10.1016/j.ydbio.2003.10.012) 14729484

[73] Miller CT, Yelon D, Stainier DY, Kimmel CB 2003 Two *endothelin 1* effectors, *hand2* and *bapx1*, pattern ventral pharyngeal cartilage and the jaw joint. Development 130, 1353–1365. (10.1242/dev.00339) 12588851

[74] Anthwal N, Joshi L, Tucker AS 2013 Evolution of the mammalian middle ear and jaw: adaptations and novel structures. J. Anat. 222, 147–160. (10.1111/j.1469-7580.2012.01526.x) 22686855 PMC3552421

[75] Couly GF, Coltey PM, Le Douarin NM 1993 The triple origin of skull in higher vertebrates: a study in quail-chick chimeras. Development 117, 409–429. 8330517 10.1242/dev.117.2.409

[76] Allin EF, Hopson JA 1992 Evolution of the auditory system in Synapsida (‘mammal- like reptiles’ and primitive mammals) as seen in the fossil record. In The evolutionary biology of hearing (eds Webster DB, Fay RR, Popper AN), pp. 587–614. New York, NY: Springer.

[77] Maier W 1987 The angular process of *Monodelphis domestica* (Didelphidae, Marsupialia) and its relation to the middle ear: an ontogenetic and evolutionary morphologic study. Gegenbaurs Morphol. Jahrb. 133, 123–161. 3569814

[78] Smith KK 1994 Development of craniofacial musculature in *Monodelphis domestica* (Marsupialia, Didelphidae). J. Morphol. 222, 149–173. (10.1002/jmor.1052220204) 7799438

[79] Muller F 1968 Transitory closures in the postnatal development of Marsupialia. Acta Anat. 71, 581–624. (10.1159/000143207) 5753480

[80] Sanchez-Villagra MR, Gemballa S, Nummela S, Smith KK, Maier W 2002 Ontogenetic and phylogenetic transformations of the ear ossicles in marsupial mammals. J. Morphol. 251, 219–238. (10.1002/jmor.1085) 11835361

[81] Aitkin L, Cochran S, Frost S, Martsi-McClintock A, Masterton B 1997 Features of the auditory development of the short-tailed Brazilian opossum, *Monodelphis domestica*: evoked responses, neonatal vocalizations and synapses in the inferior colliculus. Hear. Res. 113, 69–75. (10.1016/S0378-5955(97)00128-7) 9387986

[82] Depew MJ, Lufkin T, Rubenstein JL 2002 Specification of jaw subdivisions by *Dlx* genes. Science 298, 381–385. (10.1126/science.1075703) 12193642

[83] Depew MJ, Compagnucci C 2008 Tweaking the hinge and caps: testing a model of the organization of jaws. J. Exp. Zool. B Mol. Dev. Evol. 310, 315–335. (10.1002/jez.b.21205) 18027841

[84] Hall BK 2005 Bones and cartilage: developmental and evolutionary skeletal biology. San Diego, CA: Elsevier.

[85] Kemp TS 2005 The origin and evolution of mammals. Oxford, UK: Oxford University Press.

[86] Luo Z-X 2011 Developmental patterns in Mesozoic evolution of mammal ears. Annu. Rev. Ecol. Evol. Syst. 42, 355–380. (10.1146/annurev-ecolsys-032511-142302)

[87] Anthwal N, Chai Y, Tucker AS 2008 The role of transforming growth factor-beta signalling in the patterning of the proximal processes of the murine dentary. Dev. Dyn. 237, 1604–1613. (10.1002/dvdy.21567) 18498113

[88] Duterloo HS, Jansen HW 1969 Chondrogenesis and osteogenesis in the mandibular condylar blastema. Rep. Congr. Eur. Orthod. Soc., 109–118. 5272769

[89] Vinkka H 1982 Secondary cartilages in the facial skeleton of the rat. Proc. Finn. Dent. Soc. 78(Suppl 7), 1–137. 7184017

[90] Shibata S, Sato R, Murakami G, Fukuoka H, Rodrigues-Vazquez JF 2013 Origin of mandibular condylar cartilage in mice, rats, and humans: periosteum or separate blastema? J. Oral Biosci. 55, 208–216. (10.1016/j.job.2013.08.001)

[91] Shibata S, Suda N, Yoda S, Fukuoka H, Ohyama K, Yamashita Y, Komori T 2004 *Runx2*-deficient mice lack mandibular condylar cartilage and have deformed Meckel's cartilage. Anat. Embryol. 208, 273–280. (10.1007/s00429-004-0393-2) 15156401

[92] Fukuoka H, Shibata S, Suda N, Yamashita Y, Komori T 2007 Bone morphogenetic protein rescues the lack of secondary cartilage in *Runx2*-deficient mice. J. Anat. 211, 8–15. (10.1111/j.1469-7580.2007.00739.x) 17555546 PMC2375790

[93] Buxton PG, Hall B, Archer CW, Francis-West P 2003 Secondary chondrocyte-derived *Ihh* stimulates proliferation of periosteal cells during chick development. Development 130, 4729–4739. (10.1242/dev.00610) 12925598

[94] Solem RC, Eames BF, Tokita M, Schneider RA 2011 Mesenchymal and mechanical mechanisms of secondary cartilage induction. Dev. Biol. 356, 28–39. (10.1016/j.ydbio.2011.05.003) 21600197 PMC3130809

[95] Hall BK, Herring SW 1990 Paralysis and growth of the musculoskeletal system in the embryonic chick. J. Morphol. 206, 45–56. (10.1002/jmor.1052060105) 2246789

[96] Hall BK 1984 Developmental processes underlying the evolution of cartilage and bone. In The structure, development and evolution of reptiles (ed. Ferguson MWJ), pp. 155–176. London, UK: Academic Press.

[97] Purcell P, Joo BW, Hu JK, Tran PV, Calicchio ML, O'Connell DJ, Maas RL, Tabin CJ 2009 Temporomandibular joint formation requires two distinct hedgehog-dependent steps. Proc. Natl Acad. Sci. USA 106, 18 297–18 302. (10.1073/pnas.0908836106) 19815519 PMC2775291

[98] Shibukawa Y, Young B, Wu C, Yamada S, Long F, Pacifici M, Koyama E 2007 Temporomandibular joint formation and condyle growth require Indian hedgehog signaling. Dev. Dyn. 236, 426–434. (10.1002/dvdy.21036) 17191253

[99] Gu S et al. 2014 BMPRIA mediated signaling is essential for temporomandibular joint development in mice. PLoS ONE 9, e101000 (10.1371/journal.pone.0101000) 25093411 PMC4122352

[100] Wang Y et al. 2011 Tissue interaction is required for glenoid fossa development during temporomandibular joint formation. Dev. Dyn. 240, 2466–2473. (10.1002/dvdy.22748) 21953591 PMC3197963

[101] Jing Y, Zhou X, Han X, Jing J, von der Mark K, Wang J, de Crombrugghe B, Hinton RJ, Feng JQ 2015 Chondrocytes directly transform into bone cells in mandibular condyle growth. J. Dent. Res. 94, 1668–1675. (10.1177/0022034515598135) 26341973 PMC4681473

[102] Henson OW 1974 Comparative anatomy of the middle ear. In Handbook of sensory physiology: the auditory system (eds Keidel WD, Neff WD), pp. 39–110. Berlin, Germany: Springer.

[103] Anthwal N, Tucker AS 2012 Molecular biology of the mammalian dentary: insights into how complex skeletal elements can be shaped during development and evolution. In From clone to bone (eds Asher RJ, Müller) J, pp. 207–229. Cambridge, UK: Cambridge University Press.

[104] Aggarwal VS, Carpenter C, Freyer L, Liao J, Petti M, Morrow BE 2010 Mesodermal *Tbx1* is required for patterning the proximal mandible in mice. Dev. Biol. 344, 669–681. (10.1016/j.ydbio.2010.05.496) 20501333 PMC2917794

[105] Kist R, Greally E, Peters H 2007 Derivation of a mouse model for conditional inactivation of *Pax9*. Genesis 45, 460–464. (10.1002/dvg.20295) 17610273

[106] Sanford LP et al. 1997 TGFbeta2 knockout mice have multiple developmental defects that are non-overlapping with other TGFbeta knockout phenotypes. Development 124, 2659–2670. 9217007 10.1242/dev.124.13.2659 PMC3850286

[107] Anthwal N, Peters H, Tucker AS 2015 Species-specific modifications of mandible shape reveal independent mechanisms for growth and initiation of the coronoid. EvoDevo 6, 35 (10.1186/s13227-015-0030-6) 26568815 PMC4644282

